# A Phase II Nonrandomised Open-Label Study of Liposomal Daunorubicin (DaunoXome) in Advanced Soft Tissue Sarcoma

**DOI:** 10.1155/SRCM/2006/41080

**Published:** 2006-05-11

**Authors:** Anne McTiernan, Jeremy Whelan, Michael Leahy, Penella J. Woll, Ian Judson

**Affiliations:** ^1^Meyerstein Institute of Oncology, The Middlesex Hospital, University College London Hospitals (UCLH), London W1T 3AA, UK; ^2^St James Univesity Hospitals, Leeds Cancer Centre, Leeds LS9 7TF, UK; ^3^Academic Department of Clinical Oncology, Nottingham City Hospital, Nottingham NG5 1PB, UK; ^4^University of Sheffield, Weston Park Hospital, Sheffield S10 2SJ, UK; ^5^Sarcoma Unit, Royal Marsden NHS Trust, 203 Fulham Road, London SW3 6JJ, UK

## Abstract

Thirty four patients with advanced soft tissue sarcoma not previously treated with an anthracycline were treated with DaunoXome 100mg/m2 every 3 weeks. Thirty-three patients were evaluable for toxicity. Grade 3-4 neutropenia was seen in 20 patients (60.6%), complicated by febrile neutropenia in 2 (6.1%). Other grade 3 toxicities were rare. Among 32 patients assessable for response, one patient had a partial response, giving a response rate of 3.13% (95% confidence interval, 0.08–16.22%). Seven patients (21.9%) had stable disease, and 24 patients (75.0%) had disease progression. The median time to progression for all patients was 42 days (95% CI, 39–49) and the progression-free rate at 3 months was 12.5%. In conclusion, DaunoXome at this dose and schedule is well tolerated in patients with advanced soft tissue sarcoma, but is not associated with significant activity. Further studies at this dose and schedule cannot be recommended in this disease.

## INTRODUCTION

For patients with advanced or metastatic adult soft tissue sarcomas, the response rate to anthracycline-containing first-line chemotherapy regimens is 26%, and the median survival is just 12 months [1]. Hence there is need for more effective treatments in this group of patients. Doxorubicin appears to be the most active agent in advanced soft tissue sarcoma, with a meta-analysis of 764 patients treated with single-agent doxorubicin finding a response rate between 16% and 27% [2]. However, treatment with doxorubicin is limited by mucositis, bone marrow suppression, and cardiotoxicity. Alternative anthracyclines have efficacy in soft tissue sarcoma, but none has proved superior to doxorubicin [3, 4]. There is as yet no compelling evidence that combination chemotherapy regimens are superior to single-agent doxorubicin [5].

Liposomal anthracyclines may offer an advantage over conventional anthracycline preparations, as they have been shown selectively to enhance the delivery of drug to tumours whilst reducing the toxicity profile [6–8]. DaunoXome is a liposomal formulation of daunorubicin in which the drug is entrapped into small unilamellar vesicles (40–60 nm) composed of a 2 : 1 molar ratio of highly purified distearoyl phosphatidylcholine and cholesterol. In human pharmacokinetic studies, DaunoXome administration resulted in a greater than 100-fold increase in mean peak plasma levels compared to conventional drug, with a 36-fold increase in the area under the plasma curve [7]. Initial phase I studies in refractory solid tumours determined the maximum tolerated dose to be 100–120 mg/m^2^ every 3 weeks, with the dose-limiting toxicity being myelosuppression [8]. Severe cardiotoxicity is rare, with one study finding no cardiac toxicity in 88 patients treated with a cumulative dose between 600 and 3159 mg/m^2^ [9].

Activity with liposomal daunorubicin has been demonstrated in a range of tumours including the breast cancer [10], the childhood brain cancers [11], the non-Hodgkin's lymphoma [12], and the most notably in Kaposi's sarcoma [13, 14] for which liposomal daunorubicin is now licensed.

The aim of this study was to assess the therapeutic activity of DaunoXome as first-line treatment in advanced soft tissue sarcoma of adults. The endpoints of the study were objective response rate, duration of response, and toxicity.

## PATIENTS AND METHODS

### Eligibility criteria

Patients with histologically proven soft tissue sarcoma, and locally advanced or metastatic disease unsuitable for curative treatment with surgery or radiotherapy, were included. Patients had to have bidimensionally measurable disease with evidence of progression within 6 weeks prior to treatment. Other eligibility criteria included age ≥ 18 years; World Health Organization (WHO) performance status ≤ 2; life expectancy of 12 weeks or greater; absolute neutrophil count of ≥ 2 ×10^9^/L and platelets ≥ 100×10^9^/L; serum creatinine ≤ 140 μmol/L; serum bilirubin ≤ 1.25× upper normal limit (N); and AST ≤ 2× N (≤ 3× N in the case of liver metastases). Patients who had received prior chemotherapy for advanced disease were excluded, as were patients who had received adjuvant anthracycline therapy. A minimum of 4 weeks (8 weeks in the case of extensive prior radiotherapy) must have elapsed between the end of prior radiotherapy and entry into the protocol.

Ethics approval was granted at all participating institutions, and written informed consent was obtained from all patients in accordance with local regulatory guidelines prior to study entry.

### Study evaluations

At study entry, and prior to each course of chemotherapy, patients had a complete history and physical examination. Blood tests, including renal, liver, and bone biochemistry were performed every three weeks, together with a weekly full blood count. Left ventricular ejection fraction was evaluated at baseline and after every 2 cycles of chemotherapy. Clinical and/or radiological assessment of disease was performed within 8 days prior to the first infusion, and after every 2 cycles of chemotherapy thereafter, using the same method of evaluation.

### Treatment

DaunoXome 100 mg/m^2^ was diluted with 5% dextrose injection and infused intravenously over a minimum of 2 hours, on an outpatient basis. Cycles were repeated every 21 days, upon recovery of neutrophils to ≥ 2.0×10^9^/L. Treatment was limited to two cycles except in patients who showed unequivocal benefit from treatment with DaunoXome. In the absence of obvious disease progression after one cycle, patients were reassessed for response after 2 cycles. Patients showing an objective response, that is, complete or partial remission, received at least 2 further cycles of treatment, and continued on DaunoXome until disease progression or clinician or patient choice. Patients with minor response or disease stabilisation were also allowed to continue.

Toxicity was graded according to the National Cancer Institute–Common Toxicity Criteria (NCI-CTC), version 2.0 [15].

### Evaluation of response

Response to DaunoXome was assessed after every two cycles of chemotherapy according to the WHO criteria for clinical response [16].

### Statistical methods

Prior to the initiation of the study, it was considered that the lowest limit of therapeutic activity of interest was a response rate to DaunoXome of 20%. The Gehan two-step procedure was used to calculate the number of patients required [17]. Initially, 14 eligible patients would be recruited. If no responses were observed in these patients, the study would be terminated. Under these conditions, the probability of rejecting the treatment, if it was at least 20% effective, was less than 5%. If at least one response was observed, more patients would be added depending on the number of responses observed in order to estimate the therapeutic effectiveness with a standard error of 10%.

## RESULTS

Between July 1998 and August 2002, 36 patients from 4 centres were enrolled on the study. Two patients were subsequently found to be ineligible due to a diagnosis of gastrointestinal leiomyosarcoma in both cases. The patient characteristics for the remaining 34 eligible patients are given in Table 1.

### Treatment

A total of 83 cycles was given, with a median of 2 cycles per patient (range, 1–6). Two patients required a dose reduction to 80 mg/m^2^ for second and subsequent cycles of chemotherapy due to febrile neutropenia after the first cycle of treatment. Treatment was repeated at a median of 21 days (range, 20–28). Only 2 patients experienced treatment-related delays. One patient required a 2-day delay because of febrile neutropenia in the preceding cycle, and one patient had an allergic reaction to cycle 2 for which the infusion was discontinued, but was successfully retreated at full dose 7 days later, with antihistamine and corticosteroid prophylaxis.

### Toxicity

Thirty three patients were evaluable for assessment of toxicity. One further patient had an allergic reaction after just 10 minutes of treatment, following which the infusion was terminated and no further drug was given. This patient was therefore only eligible for assessment of hypersensitivity reactions.

There were no deaths attributable to the study drug. The principal haematological toxicity was leukopenia, with 20 patients (60.6%) experiencing grade 3-4 neutropenia, complicated by febrile neutropenia in 2 patients (6.1%). Grade 3-4 thrombocytopenia and anaemia were rare, occurring in just 2 patients (6.1%) and 1 patient (3.0%), respectively. The most frequent nonhaematological toxicity was nausea, occurring in 24 patients (72.7%), although this was only severe (grade 3) in 4 patients. Other nonhaematological grade 3 toxicities included infection (1 patient); lethargy (2 patients); diarrhoea (1 patient); and vomiting (1 patient). Alopecia was observed in 9 patients, but this was mild in all cases. There were no reported cases of cardiac toxicity.

Hypersensitivity reactions occurred in 3 of the 34 patients (8.8%). Two patients experienced moderate reactions, with facial flushing and back pain. Both patients were treated with intravenous antihistamine and corticosteroids and were able to complete the treatment as planned, with a slower rate of infusion. One further patient with a history of hypertension and two prior transient ischaemic attacks experienced chest pain after 10 minutes of the infusion. The infusion was discontinued and intravenous antihistamine and steroid were administered. An ECG was performed which was abnormal hence the patient was admitted for observation. The chest pain resolved after 5 hours and subsequent tests showed no evidence of myocardial infarction. The patient was discharged home 2 days later. No further drug was administered and the patient was withdrawn from the study. It is not clear whether or not this episode was related to study drug treatment.

### Response

Thirty two patients were evaluable for response to DaunoXome. One patient with a retroperitoneal sarcoma died of a perforated duodenal ulcer, 19 days after cycle 1. A causal relationship with the drug was deemed possible, but not proven. One further patient discontinued treatment after 10 minutes due to hypersensitivity, and received no further treatment.

Among the 32 eligible patients, one partial response was observed in the first 14 patients and the trial was expanded. In total, 36 patients were treated owing to the lack of alternative treatments available for some of these patients and the level of disease stabilisation observed. The observed response was in a patient with malignant fibrous histiocytoma, giving a response rate of 3.13% (95% confidence interval (Cl), 0.08–16.22%). The duration of this response was 77 days. Seven patients (21.9%), all of whom had progressive disease in the 6 weeks prior to treatment, had disease stabilisation in response to DaunoXome for a median of 87 days (range, 65–384). The median time to progression for all patients was 42 days (95% Cl, 39–49) and progression-free survival at 3 months was 12.5%.

#### Subsequent treatment and survival

Eighteen patients received further treatment, 14 with chemo-therapy, principally with ifosfamide and/or doxorubicin-based chemotherapy. One patient, with a partial response to DaunoXome, achieved a further partial response to single-agent ifosfamide. No other objective responses were observed to second-line chemotherapy. The median overall survival from the start of DaunoXome was 197 days (95% Cl, 144–250).

## DISCUSSION

Although doxorubicin would appear to be among the most active agents in soft tissue sarcoma, single-agent daunorubicin has also been demonstrated to have activity in this disease. In a review that included clinical trials, single-investigator reports, and the files of the Investigational Drug Branch of the National Cancer Institute, a response rate of 20% (utilizing the response criteria of the Southwest Oncology Group) was found in 54 patients treated with single-agent daunorubicin, at varying doses and schedules [18]. Although these reports are subject to selection bias, the response rate of 3% to liposomal daunorubicin observed in this study is therefore disappointing, given the higher response rates reported with conventional anthracyclines.

It is possible that our low response rate was due to a negative patient selection bias, whereby the perceived low toxicity profile of DaunoXome may have led to the selection of patients with more indolent tumours that were less likely to respond to treatment, or patients with poorer performance status. In a study which combined liposomal daunorubicin 100 mg/m^2^ with ifosfamide 5 g/m^2^ given at four-weekly intervals as first-line treatment for advanced soft tissue sarcoma, Deckert et al found a response rate of 31.4%, with 11 of 35 patients achieving a partial response [19]. The median progression-free survival in their study was 10 months.

Although some patients experienced disease stabilisation, the progression-free rate at 3 months was only 12.5%. This does not equate with activity by the criteria reported by van Glabbeke et al [20]. In their analysis, active agents against soft tissue sarcoma gave progression-free rates of 30%–56% at 6 months and a progression-free rate of < 20% at 3 months indicated lack of activity.

Toxicity of DaunoXome was tolerable. Only 2 patients experienced febrile neutropenia and other grade 3 toxicities were rare. Although mucositis was observed in 8 patients (24%), this was mild to moderate in all cases. There were no reported cases of cardiotoxicity, although this was unlikely to be observed, given that the number of cycles administered ranged from 1 to 6 (median 2), which would not approach the cardiotoxic dose of the anthracycline.

In conclusion, single-agent DaunoXome at this dose and schedule does not appear to be active in patients with advanced soft tissue sarcoma, and cannot be recommended for further study in this disease.

## Figures and Tables

**Table 1:** Patient characteristics (*n* = 34).

	No (%)

Male : Female	15 : 19
Median age:	59 years (range, 28–80)
WHO performance status	
0	6 (17.6)
1	23 (67.6)
2	5 (14.7)
Histological subtype	
Leiomyosarcoma	14 (41.2)
Unclassified	6 (17.6)
Liposarcoma	3 (8.8)
Angiosarcoma	2 (5.9)
Synovial sarcoma	2 (5.9)
Malignant fibrous histiocytoma	2 (5.9)
Malignant peripheral nerve sheath tumour	2 (5.9)
Other*	3 (8.8)
Primary site	
Thigh	8 (23.5)
Buttock	5 (14.7)
Uterus	4 (11.8)
Intra-abdominal	4 (11.8)
Retroperitoneal	2 (5.9)
Knee or lower leg	5 (14.7)
Other^†^	6 (17.6)
Site of disease at start of treatment	
Primary site/recurrence	13 (38.2)
Lung	22 (64.7)
Soft tissue	5 (14.7)
Liver	6 (17.6)
Skin	3 (8.8)
Lymph	3 (8.8)
Bone	2 (5.9)
No of sites of disease	
1	20 (58.8)
2	9 (26.5)
≥ 3	5 (14.7)
Prior treatment	
Surgery	—
Curative	25 (73.5)
Palliative	1 (2.9)
Radiotherapy	13 (38.2)
Chemotherapy	0
Median time from diagnosis to start of DanuoXome	10 months (range, 0–147)
Median time from last radiotherapy administration to start of DaunoXome (*n* = 13)	8 months (range, 2–39)


*Clear cell (1), haemangiopericytoma (1), pleiomorphic rhabdomyosarcoma (1).

^†^Anterior/posterior mediastinal (2), paravertebral (1), shoulder (1), upper arm (1), renal (1).
